# The NAT10/ac4C axis: bridging RNA epigenetics to human diseases

**DOI:** 10.3389/fcell.2026.1905147

**Published:** 2026-10-28

**Authors:** Ruiqi Li, Xuemei Gan, Lan Huang, Xin Zhang, Luyu Wen, Yang Cao, Junyu Zhou, Fei Xie, Hong Liu

**Affiliations:** 1 School of Clinical Medicine, Southwest Medical University, Luzhou, Sichuan, China; 2 Department of Hepatology, Dazhou Central Hospital, Dazhou, Sichuan, China; 3 Department of Hepatobiliary Surgery, Dazhou Central Hospital, Dazhou, Sichuan, China

**Keywords:** N4-acetylcytidine, cancer, epigenetics, NAT10, RNA modification

## Abstract

N4-acetylcytidine (ac4C) stands as the sole RNA acetylation mark identified to date in eukaryotes, dynamically installed by N-acetyltransferase 10 (NAT10). This modification has been linked to mRNA stability and translation, and its dysregulation is increasingly implicated in human pathologies. This review synthesizes current knowledge on the discovery, distribution, and deposition of ac4C, with a focus on its writer, NAT10. We discuss the roles of ac4C in safeguarding tRNA decoding fidelity, modulating RNA metabolism, and cellular stress responses. Furthermore, we systematically examine the expanding landscape of NAT10/ac4C dysfunction in cancer, cardiovascular diseases, metabolic disorders, infectious diseases, immune-mediated inflammatory conditions, and nervous system diseases. By examining current limitations, we propose key directions for future investigation, including the development of advanced detection tools and targeted therapeutic strategies. We suggest that the NAT10/ac4C axis may constitute a signaling cascade hub, maintaining homeostasis under physiological conditions and translating persistent stress signals into disease-specific phenotypes under pathological conditions. This framework will provide a conceptual basis for translating ac4C biology into clinical applications.

## 1 Introduction

Among the RNA modifications known today, N4-acetylcytidine (ac4C) has recently captured significant attention as the sole acetylation mark identified on eukaryotic RNAs. It was first discovered in tRNA (Zachau et al., 1966) and 18S rRNA (Thomas et al., 1978), and has been reported in mRNA and other non-coding RNAs. Previous studies have shown that ac4C is involved in regulating RNA stability, translation efficiency, and various biological processes, including metabolism and cell division (Lou et al., 2023). In eukaryotic cells, N-acetyltransferase 10 (NAT10) is the only known enzyme that catalyzes ac4C formation in RNAs. This process requires acetyl-CoA and ATP hydrolysis. With the development of high-throughput sequencing technologies, the clinical relevance of the NAT10/ac4C axis has been preliminarily validated in multiple diseases. This axis exhibits disease-type-dependent patterns: in synovial tissue from patients with rheumatoid arthritis and in bladder cancer tissue, both NAT10 and ac4C are significantly elevated (Liu D. et al., 2024; Xie et al., 2023); conversely, in bone tissue from patients with osteoporosis and in CD4^+^ T cells from patients with systemic lupus erythematosus, both are significantly reduced (Guo et al., 2020; Yang et al., 2021). This bidirectional expression pattern suggests that the axis is not a unidirectional driver in pathology but rather produces distinct functional outputs depending on cell type and disease context. Notably, ac4C readers and erasers have not yet been conclusively identified, limiting our understanding of this axis. Here, we propose that the NAT10/ac4C axis exhibits functional plasticity across different cellular signaling contexts, contributing to homeostasis under physiological conditions while driving disease progression under pathological stress, and may thus serve as a cross-disease signaling cascade hub. Through systematic reviewing of its multifaceted roles in cancer, cardiovascular diseases, metabolic disorders, infectious diseases, immune-mediated inflammatory conditions, and nervous system diseases, we aim to provide an integrative framework for understanding how ac4C exerts differential regulatory functions in diverse disease settings.

## 2 Biogenesis and detection of ac4C

### 2.1 Chemical structural characteristics of ac4C

Ac4C involves the attachment of an acetyl group (-CO-CH_3_) to the nitrogen atom at the N4 position of cytidine. The nitrogen atom of the N4 amino group is covalently bonded to the carbonyl carbon atom of the acetyl group, forming an N-acetylated structure (Figure 1). This structure is a key distinguishing feature of ac4C among other RNA modifications (Schiffers and Oberdoerffer, 2024).

**FIGURE 1:**
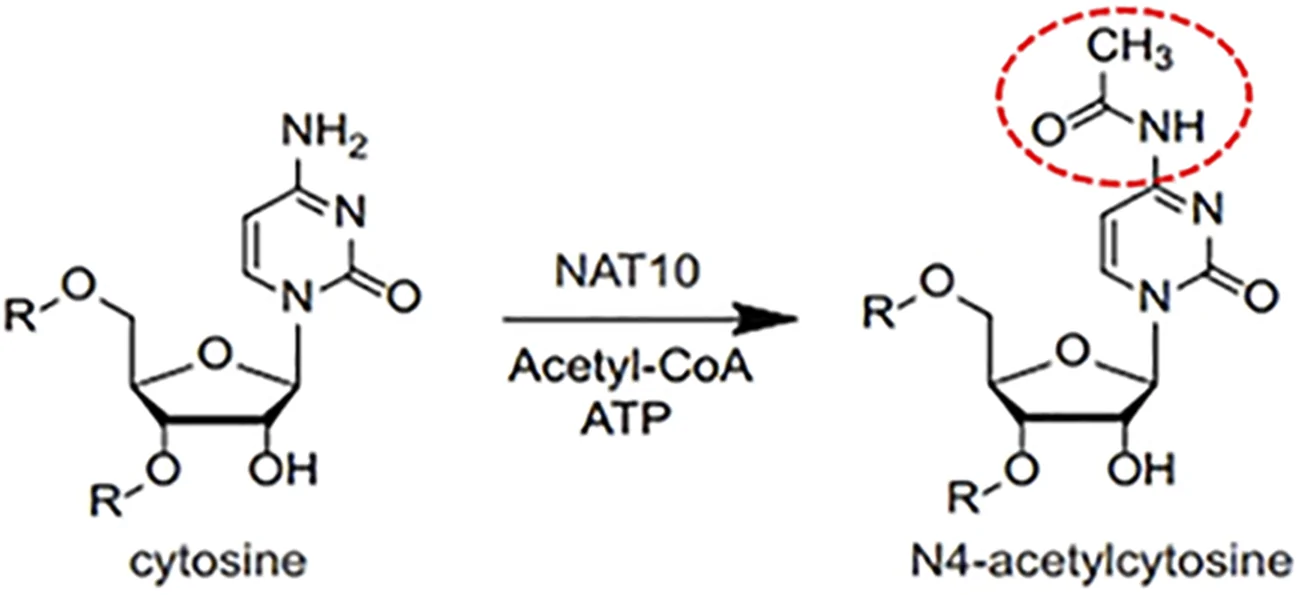
Chemical structure of ac4C. The acetyl group attached to the N4 position of cytidine is highlighted.

### 2.2 ac4C on different RNAs

#### 2.2.1 ac4C on tRNA

Ac4C primarily targets specific sites that significantly impact tRNA stability and function, such as the anticodon loop, assisting in proper tRNA folding and ensuring precise codon-anticodon pairing. In the 1960s, ac4C was first reported in yeast tRNASer (Zachau et al., 1966). Using comparative ultraviolet absorption spectroscopy, Ohashi et al. (1972) identified ac4C at the first anticodon position of tRNAMet in *Escherichia coli* (ac4C34, position 34 of the tRNA). In the cytoplasm of eukaryotic cells, however, ac4C is located at position 12 of both tRNASer and tRNALeu (Kowalski et al., 1971; Kruppa and Zachau, 1972).

#### 2.2.2 ac4C on rRNA

Ac4C is primarily located in the small ribosomal subunit of rRNA. In 1978, Thomas et al. (1978) first revealed the presence of ac4C in the 18S rRNA of eukaryotic cells and proposed that ac4C is relatively conserved in the small ribosomal subunits of eukaryotes. In 1993, ac4C was discovered in the 5S rRNA of hyperthermophilic archaea (Bruenger et al., 1993). In 2015, to precisely locate the position of the ac4C modification on 18S rRNA within the small ribosomal subunit, Sharma et al. (2015) quantified ac4C by reverse-phase high-performance liquid chromatography and mapped this modification to the helix 34 (H34) and helix 45 (H45) regions of the 18S rRNA secondary structure.

#### 2.2.3 ac4C on mRNA

Early studies of ac4C focused on its roles in tRNA and rRNA. In 2018, Arango et al. reported the detection of ac4C in human mRNAs with sites enriched within coding sequences and an abundance gradient decreasing from the 5′ to 3′ ends, suggesting a potential role in translation regulation (Arango et al., 2018). In 2019, ac4C was identified in yeast mRNA. And Rra1, a NAT10 homolog, was shown to be responsible for its incorporation (Tardu et al., 2019). However, subsequent studies have raised questions about the abundance and transcriptome-wide distribution of mRNA ac4C. These ongoing debates are further discussed in Section 6.1.1.

#### 2.2.4 ac4C on other RNAs

In addition to tRNA, rRNA, and mRNA, ac4C is also distributed in other non-coding RNAs, such as lncRNA and pri-miRNA. Recent studies indicate that the ac4C modification stabilizes the lncRNA CD2BP2-DT, which in turn drives the liquid–liquid phase separation of YBX1, thereby stabilizing *CDK1* mRNA and promoting the proliferation of breast cancer cells (Wang H. et al., 2025). The biogenesis of miRNAs is also associated with ac4C. Zhang H. et al. (2024) found through *in vivo* and *in vitro* experiments that NAT10 binds to pri-miRNA containing an RNA stem-loop hairpin structure. Together with THUMPD1, it mediates ac4C formation, enhances the interaction between pri-miRNA and DGCR8, and promotes the processing of pri-miRNA into precursor miRNA.

### 2.3 Synthesis pathway of ac4C

RNA acetyltransferase is necessary for the production of ac4C. Under ATP-powered circumstances, this enzyme first identifies specific sequences on the target RNA before moving the acetyl group from acetyl-CoA (the typical acetyl donor) to the fourth nitrogen atom of cytidine. Because of its high specificity, this enzymatic alteration guarantees accurate ac4C deposition. The following three categories comprise the relevant enzymes.

#### 2.3.1 TmcA-type RNA acetyltransferase

In 2008, Ikeuchi et al. (2008) discovered tRNAMet cytidine acetyltransferase (TmcA) in *E. coli*. This enzyme possesses two catalytic domains: a C-terminal acetyltransferase domain belonging to the GNAT family and an N-terminal ATPase domain containing a Walker motif. In the presence of acetyl-CoA and ATP/GTP, TmcA specifically interacts with tRNAMet to catalyze the ac4C formation at the wobble position of *E. coli* tRNAMet. This prevents misreading of the isoleucine codon AUA and stabilizes the tertiary structure of the tRNA molecule.

#### 2.3.2 NAT10/Kre33-type RNA acetyltransferase

In 2014, Ito et al. (2014b) first identified the yeast protein Kre33 (also known as Rra1/Rra1p, encoded by KRE33) as the cytidine acetyltransferase responsible for ac4C1773 in 18S rRNA. It is a modification essential for the processing of 23S pre-rRNA into mature 18S rRNA. In the same year, a study further demonstrated that NAT10, the human ortholog of Rra1p, catalyzes ac4C1842 in human 18S rRNA (Ito et al., 2014a). Subsequently, Sharma et al. extended this function to tRNA, showing that human NAT10 and its yeast counterpart Rra1p form complexes with their respective partners—THUMPD1 and TAN1—to mediate ac4C acetylation at position 12 of leucine and serine tRNAs in both species (Sharma et al., 2015). Later work revealed that box C/D snoRNAs (snR4 and snR45) interact with Rra1p to guide ac4C deposition in 18S rRNA (Sharma et al., 2017). In 2018, Arango et al. proposed that NAT10 serves as the primary ac4C writer on mRNA (Arango et al., 2018). More recently, Jiang et al. identified PCBP1/2 and TDP43 as mRNA-binding adaptors for NAT10, influencing ac4C site distribution on mRNA (Jiang et al., 2024). Given its central role in multiple RNA classes, NAT10 is tightly regulated. Its dysregulation has been linked to various diseases, making it a potential target for small-molecule inhibitors such as Remodelin.

#### 2.3.3 TmcAL-type RNA acetyltransferase

In 2018, Taniguchi et al. (2018) revealed that TmcAL functions as a novel enzyme responsible for ac4C34 formation. This enzyme neither uses acetyl-CoA as a cofactor nor contains a helicase domain or GNAT domain. This enzyme activates acetate ions to form acetyladenylate (acetyl-AMP, a high-energy intermediate), thereby generating ac4C through a mechanism similar to tRNA aminoacylation.

### 2.4 Regulatory mechanisms of the NAT10/ac4C axis

The landscape of ac4C modification is primarily shaped by its writer, NAT10. While readers and erasers are well established for other modifications like N6-methyladenosine (m6A), their identity for ac4C remains a mystery in this field. Proteins such as NOP58 (Kudrin et al., 2024), EEF2 (Liu H. et al., 2024), and MYBBP1A (Wang H. et al., 2026) have been proposed as candidate readers that bind ac4C-containing RNAs. Similarly, the deacetylase SIRT7 has been suggested as a potential eraser (Xu et al., 2021), but whether it acts on mRNA ac4C has not been demonstrated. Identifying and validating genuine ac4C readers and erasers is a priority, because they are essential for understanding ac4C’s diverse downstream effects. In the absence of known erasers and readers, the regulation of ac4C is currently understood as a multi-level network centered on NAT10 (Figure 2).

**FIGURE 2:**
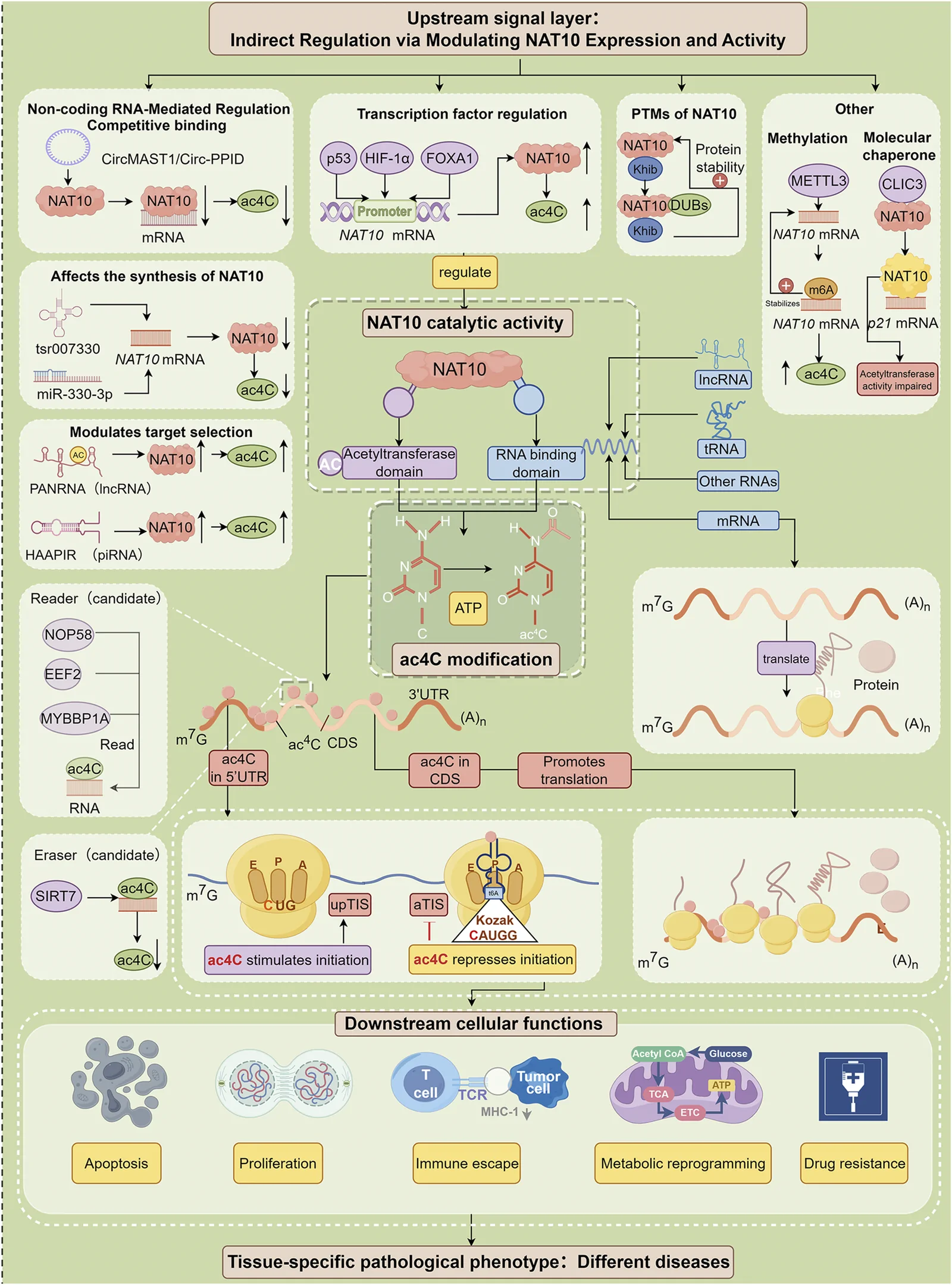
Multi-level regulatory network of the NAT10/ac4C axis. Schematic representation of the NAT10-ac4C axis in five layers: Upstream signal layer → NAT10 catalytic activity → ac4C modification (diverse RNA substrates: mRNA, lncRNA, tRNA, etc.) → downstream cellular functions (position-dependent effects of ac4C at 5′UTR vs. CDS) → tissue-specific pathological phenotypes.

#### 2.4.1 Direct regulation by NAT10

NAT10 directly regulates ac4C deposition by binding target RNAs and catalyzing the modification through its catalytic domain.

#### 2.4.2 Indirect regulation via modulating NAT10 expression and activity

##### 2.4.2.1 Non-coding RNA-mediated regulation

Non-coding RNAs such as tsRNA, circRNA, lncRNA, piRNA, and miRNA can serve as upstream regulatory molecules of NAT10, indirectly modulating ac4C. Some non-coding RNAs competitively bind to NAT10, thereby blocking its function. In cervical cancer, circMAST1 binds to NAT10 to promote *YAP* mRNA degradation, inhibiting tumor growth and metastasis (Zhang C. et al., 2024). Similarly, circ-PPID reduces ac4C levels in *HER2* mRNA by binding to NAT10 and blocking its interaction with *HER2* mRNA (Wang S. et al., 2024). Certain non-coding RNAs show their effects by influencing NAT10’s synthesis. Research indicates that tsr007330 can bind to *NAT10* mRNA, downregulating NAT10 expression and improving myocardial fibrosis (Hao et al., 2024). Similarly, miR-330-3p binds to the 3’UTR of *NAT10* mRNA, significantly suppressing NAT10 expression (Wu J. et al., 2026). Additionally, several non-coding RNAs bind NAT10 and modulate its target selection. Yan et al. (2023) discovered that NAT10’s identification of its target *IFI16* mRNA is improved when PAN RNA (lncRNA) is ac4C-modified. Similarly, HAAPIR (piRNA), acting as a heart-apoptosis-associated piRNA, promotes NAT10 binding to *Tfec* mRNA, thereby upregulating the NAT10/ac4C/*Tfec*/*Bik* signaling axis (Wang et al., 2022).

##### 2.4.2.2 Transcription factor regulation

Several transcription factors directly bind to the NAT10 promoter to initiate its transcription. In ischemia/reperfusion (I/R) injury, p53 serves as a transcriptional activator to upregulate NAT10, triggering a positive feedback loop involving Mybbp1a (Qu et al., 2024). Similarly, HIF-1α (Sun et al., 2025) and FOXA1 (Zhu A. et al., 2025) have been shown to bind the NAT10 promoter directly, upregulating its expression in the context of cancer progression.

##### 2.4.2.3 Post-translational modifications of NAT10

Multiple studies have highlighted the critical role of post-translational modifications (PTMs) in regulating NAT10 expression levels, including 2-hydroxyisobutyrylation (Khib). Khib modification at lysine 823 enhances NAT10 stability by strengthening interactions with deubiquitinating enzymes (DUBs), upregulating the NAT10/ac4C/*NOTCH3* signaling axis (Liao et al., 2023). NAT10 also undergoes autoacetylation (Cai et al., 2017), phosphorylation (Zhang et al., 2014), and poly(ADP-ribosyl)ation (Liu et al., 2022). However, additional research is necessary to determine whether these changes indirectly affect ac4C levels.

##### 2.4.2.4 Other regulatory mechanisms of NAT10

One typical methylation modification that can upregulate NAT10 is m6A. By binding to the 3’UTR of *NAT10* mRNA, the m6A methyltransferase METTL3 stabilizes *NAT10* mRNA and promotes the production of ac4C (Jin et al., 2024). Chloride intracellular channel 3 (CLIC3) may also function as a regulator of NAT10, with its GST N-terminal domain binding to the N-acetyltransferase domain of NAT10. It is thought that CLIC3 may act as a molecular chaperone, modulating the role of NAT10 in bladder cancer by changing its spatial configuration (Shuai et al., 2024).

### 2.5 Technologies for ac4C profiling and quantification

In recent years, a variety of detection technologies have been developed. Different methods have their own advantages and disadvantages in terms of operating principles, resolution, specificity, and scope of application. The choice of technology depends on the specific research objectives. Supplementary Table S1 is available for quick reference.

#### 2.5.1 Antibody-based sequencing

Acetylated RNA immunoprecipitation sequencing (acRIP-seq) is the most widely used technique for detecting ac4C. Anti-ac4C antibodies specifically bind to ac4C-containing RNA fragments. The antigen-antibody complex is captured using Protein A/G magnetic beads, and the enriched RNA is then subjected to high-throughput sequencing. This method requires relatively large amounts of starting RNA, which limits its use when sample quantities are scarce. However, its resolution is limited to regions of 100–200 nucleotides and cannot reach single-base precision. More critically, anti-ac4C antibodies may react with other acetylation modifications or structural analogs, resulting in false-positive signals. Furthermore, the strength of the enrichment signal does not directly reflect the modification ratio. Highly expressed transcripts, even if modified at a very low rate, will be captured more frequently by the antibody due to their large total RNA quantity, leading to an inflated signal. Therefore, acRIP-seq data must be interpreted with caution, and key sites should be validated using orthogonal methods (Schiffers and Oberdoerffer, 2024). To address the issue of insufficient sample size, researchers have recently developed ac4C LACE-seq, a low-input ac4C sequencing method based on antibody enrichment and linear amplification. This method detects ac4C at the peak level by exploiting reverse transcriptase termination at modified sites, combined with linear amplification for signal enhancement. It works on as few as 50 oocytes and outperforms acRIP-seq in sensitivity and signal intensity. The two methods are complementary: acRIP-seq is suitable for regional screening of routine samples, while ac4C LACE-seq is suitable for precious samples (Chen et al., 2025).

#### 2.5.2 Chemical reduction-based method

N4-acetylcytidine sequencing (ac4C-seq)/Reduction of ac4C to Tetrahydro-ac4C followed by sequencing (RedaC:T-seq) uses NaBH_3_CN/NaBH_4_ to reduce ac4C, causing C→T mismatches during reverse transcription, thereby localizing ac4C-modified sites at single-base resolution. The limit of detection depends on sequencing depth and RNA integrity. This method effectively avoids common issues such as cross-reactivity and antibody bias. But it has several limitations as well. Reduction efficiency is highly sensitive to factors such as pH and temperature; variations in experimental conditions can lead to inconsistent results. During reverse transcription, reduction products may cause polymerase termination, resulting in the loss of some signals. Furthermore, limited chemical conversion efficiency and complex secondary structures of RNA (which may embed ac4C within the molecule, thereby hindering access by chemical reagents) can both lead to an underestimation of ac4C abundance. It requires a high initial sample quantity and is not suitable for precious samples such as oocytes and embryos (Schiffers and Oberdoerffer, 2024). As shown, ac4C-seq/RedaC:T-seq is the more reliable approach for single-base resolution studies. Researchers could use acRIP-seq for initial screening and ac4C-seq/RedaC:T-seq for precise localization.

#### 2.5.3 Liquid chromatography–tandem mass spectrometry

Through chromatographic separation and mass spectrometric analysis, Liquid chromatography-tandem mass spectrometry (LC-MS/MS) can directly identify and quantify the ac4C nucleoside in RNA degradation products. This method performs well when analyzing RNAs with high ac4C content, such as tRNA and rRNA. However, it faces multiple challenges in mRNA detection: the abundance of ac4C in mRNA is extremely low, making it difficult to detect with conventional sensitivity; residual rRNA contamination can severely interfere with results, as ac4C levels in rRNA are far higher than in mRNA, and even trace amounts of residual rRNA can lead to a significant overestimation of ac4C levels; ac4C itself is chemically unstable, with approximately 25% loss occurring during 18 h of storage at pH 7 (Sinclair et al., 2017); and LC-MS/MS cannot provide information on the specific transcript or nucleotide position where ac4C is located. Therefore, LC-MS/MS is better suited for the absolute quantification of specific ac4C sites or for orthogonal validation.

#### 2.5.4 Nanopore sequencing

As RNA molecules pass through biological nanopores, different bases and their chemical modifications trigger characteristic current disturbances. Analyzing these signals allows simultaneous determination of both the sequence and its modification status. This gives nanopore sequencing a unique edge: it can detect multiple RNA modifications at the same time, achieving single-molecule, single-base resolution. In recent years, many tools have been developed, such as modCnet (Wu et al., 2025) and DirectRM (Zhang et al., 2025). However, this approach still faces several challenges. Sequencing accuracy is highly dependent on the quality of the training data and the model’s ability to generalize. The lack of unified standards across tools makes it difficult to compare results directly. Low-abundance signals are easily drowned out. Clustered or overlapping modifications produce mixed signals that are difficult to resolve. Overall, nanopore technology remains in a stage of ongoing development.

#### 2.5.5 Computational prediction

Computational prediction, typically built on machine learning or deep learning, predicts ac4C modification sites by learning from known sequence features. It is high-throughput and cost-effective, making it useful for narrowing down experimental targets. On specific datasets, this method also achieves strong predictive performance. Recent models like CapsBAM-ac4C (Shi et al., 2026) and TBC-ac4C (Zhang S. and Zhang, 2026) can both effectively predict modification sites. But it still faces the following limitations. Since the model training data is primarily derived from acRIP-seq, false positives and systematic biases exist, causing the predicted results to deviate from the true ac4C values. The models are also largely confined to the human transcriptome, with limited training data available for other species. Most existing models rely on primary sequence features, ignoring regulatory inputs like RNA secondary structure, NAT10 accessibility, and subcellular localization (Shang et al., 2026). Without these layers, current predictions fall short of capturing ac4C’s true *in vivo* behavior. So any candidate sites they yield still require experimental verification.

#### 2.5.6 Single-cell assay

FMPLA is a single‐cell in situ imaging approach for ac4C-modified RNA (Wang Q. et al., 2025). The workflow starts with fluorine-based metabolic labeling of newly formed ac4C on nascent RNA, followed by the introduction of two adjacent probes—one recognizing the tag, the other hybridizing to the target sequence. Upon binding, the probes trigger rolling-circle amplification; molecular beacons then bind the resulting amplicon and produce a fluorescent signal. This technology provides information on the subcellular distribution and abundance of ac4C at single-cell resolution and is suitable for dynamic monitoring. However, the assay detects only newly synthesized ac4C, leaving pre-existing modifications invisible. Probe design depends on known target sequences, and adjacent probe binding requires suitable spatial proximity to trigger ligation and amplification. Although the method can achieve sensitivity at the single-molecule level, it has relatively limited throughput. Moreover, quantitative accuracy is influenced by imaging conditions and analysis algorithms. Currently, the technique is best suited for studying intercellular heterogeneity, subcellular localization, and dynamic changes. In contrast, direct single-cell sequencing of ac4C has not yet been achieved; existing studies primarily rely on single-cell RNA sequencing combined with ac4C-related genetic markers to make indirect inferences.

## 3 Core cellular functions of the NAT10/ac4C axis

This paper proposes a regulatory cascade for the NAT10/ac4C axis under physiological conditions in mammals. Upstream regulatory layer: cellular signals regulate NAT10 expression and activity; Catalytic layer: NAT10 catalyzes ac4C deposition on target RNA; RNA layer: ac4C modification has been reported to influence the stability and translation efficiency of target RNA; Signal transduction layer: proteins encoded by the modified RNA alter cellular signaling networks; Cellular layer: changes in signaling ultimately drive cell proliferation and other cellular responses.

### 3.1 RNA layer: direct regulation of RNA molecules by ac4C modification

At the RNA level, ac4C modification has been most clearly established in tRNA, where it influences decoding accuracy. In mRNA, it has been reported to affect translation efficiency and subcellular distribution. Notably, this section describes the RNA layer of the NAT10/ac4C axis, including NAT10 homologs across species, such as Kre33 in yeast and TmcA in *E. coli*.

#### 3.1.1 ac4C assists in maintaining tRNA decoding fidelity

tRNA ac4C maintains decoding fidelity by correctly recognizing codons through altered interactions with AUA/AUG. Research suggests that during *in vitro* peptide synthesis, the tRNA anticodon’s ac4C regulates its binding to the AUG codon, avoiding misreading of the isoleucine codon AUA (Stern and Schulman, 1978). The responsible enzyme was later identified as TmcA, a NAT10 homolog in *E. coli*. The intramolecular hydrogen bond of ac4C’s N-acetyl group stabilizes the “proximal” orientation, enabling interaction with the third base of AUG/AUA. Conversely, the “distal” orientation prevents incorrect decoding of the isoleucine AUA codon by inhibiting this tendency (Kumbhar et al., 2013). Of note, this fidelity-related function is context-specific to tRNA and should not be generalized to mRNA.

#### 3.1.2 ac4C maintains tRNA stability and high thermal tolerance in cells

In addition to playing a role in codon recognition, ac4C modification also imparts tRNA with increased stability and thermal tolerance. The value of ac4C and TAN1 in maintaining the stability of mature tRNASerCGA was confirmed by Johansson and Byström (2004), who discovered decreased amounts of ac4C and tRNASerCGA in *Saccharomyces cerevisiae* mutants lacking the *TAN1* gene. The catalytic enzyme was later shown to be Kre33 (the homolog of mammalian NAT10), which together with TAN1 forms the ac4C modification. Kawai et al. (1991) utilized nuclear magnetic resonance proton spectroscopy to demonstrate that the ac4C modification combined with 2′-O-methylation in tRNA from extremely thermophilic archaea stabilizes the C3′-endo form, inducing “conformational rigidity” that maintains the conformation stability of specific tRNA sites under extremely high temperatures.

#### 3.1.3 ac4C regulates mRNA translation efficiency

Unlike its well-established role in safeguarding decoding fidelity on tRNA, the functional relevance of ac4C on mRNA, should it exist, has been principally linked to translational regulation rather than codon-anticodon accuracy. Early studies have proposed that ac4C regulates translation in a position-dependent manner, with 5′UTR and CDS modifications affecting initiation and elongation, respectively. In the CDS, ac4C has been suggested to enhance base-pairing stability between codons and anticodons, thereby promoting translation elongation (Arango et al., 2018). Within the 5′ UTR, ac4C is reportedly enriched near the start codon, where it is thought to modulate translation initiation (Arango et al., 2022). Mechanistically, ac4C in the 5’ UTR is proposed to promote ribosome engagement at upstream initiation sites, whereas ac4C itself disrupts Kozak sequence–initiator tRNA hydrogen bonding, thereby attenuating recognition of the canonical downstream start codon. Collectively, these observations suggest a model in which ac4C modulates translation according to regional mRNA context. However, it must be emphasized that the entirety of this functional framework rests upon the presumptive existence of ac4C on mRNA—a premise that remains contested and is itself the subject of ongoing debate (see Section 6.1.1). Thus, while the proposed mechanisms offer valuable conceptual foundations, this model should be interpreted within the bounds of current technical uncertainties.

#### 3.1.4 ac4C affects mRNA stability

Changes in translation efficiency are often accompanied by co-regulation of mRNA stability, and together influence the total protein output. In this context, ac4C has been proposed to enhance mRNA resistance to ribonuclease-mediated decay and prolong transcript half-life. Arango et al. reported that ac4C-modified mRNAs exhibited extended half-lives, particularly in transcripts with CDS-localized ac4C (Arango et al., 2018). Conversely, Zhu et al. (2021) suggested that this modification may also promote *Gremlin 1* mRNA degradation through a 3′UTR-dependent mechanism, though the details are unclear. Notably, these observations derive from experiments that presume the presence of ac4C on mRNA, a premise that has yet to be confirmed (see Section 6.1.1). Until this question is settled, conclusions regarding ac4C-mediated mRNA stability must remain tentative.

#### 3.1.5 ac4C-mediated RNA localization

The functions described above primarily determine the amount of protein produced, while the subcellular distribution of ac4C RNA constitutes another dimension of regulation. ac4C-modified mRNA is significantly enriched in stress granules (approximately 4.2-fold relative to poly(A)-selected mRNA) and can mediate the recruitment of the NOP58 protein to stress granules. Both processes depend on ac4C (Kudrin et al., 2024). However, NAT10 knockout does not affect the formation of stress granules themselves, suggesting that ac4C primarily participates in the distribution of internal components rather than structural assembly. In addition, ac4C-modified RNA can redistribute in response to DNA damage signals. Svobodová Kovaříková et al. (2023) observed that ac4C RNA accumulates specifically in regions of UV-induced intracellular DNA damage, potentially facilitating repair factor access to the damaged sites by promoting local chromatin decondensation. This ac4C modification is PARP-dependent and independent of NAT10.

### 3.2 Cellular layer

The aforementioned changes at the RNA level do not represent the endpoint of the NAT10/ac4C regulatory axis. At the translational level, ac4C in tRNA contributes to decoding fidelity. In contrast, its effects on mRNA stability and translation efficiency—though mechanistically less defined—have been suggested to modulate the protein output of specific genes, with potential implications for RNA subcellular distribution as well. These molecular events are thought to converge at the cellular level. What they orchestrate, ultimately, are fundamental processes like cell division, differentiation, development, and stress responses.

#### 3.2.1 ac4C regulates the cell division cycle and cell differentiation

During oocyte meiosis, oocyte-specific knockout of NAT10 results in decreased ac4C modification and reduced cyclin B1 protein levels, while mRNA abundance is not significantly altered, indicating that ac4C drives meiosis by enhancing the translational efficiency of cyclin B1 (Chen et al., 2025). During mitosis, the direct relationship between ac4C and cyclin mRNA remains unclear. Currently, NAT10’s regulation of the cell cycle cannot be attributed to ac4C alone; therefore, the direct association between ac4C and cyclin requires further validation. Additionally, ac4C can contribute to cell differentiation by influencing cell fate decisions. Wang et al. (2023) demonstrated that defects in *Nop2* mRNA ac4C modification inhibit the differentiation of blastomeres into trophectoderm (the first fate decision) by affecting the downstream transcription factor CDX2, thereby disrupting the transition from morula to blastocyst stage in mice.

#### 3.2.2 ac4C affects germ cell development

From oogenesis and spermatogenesis to embryonic differentiation, ac4C plays crucial roles at every stage. Lin et al. (2022) found that ac4C regulates oocyte maturation by stabilizing *OGA* mRNA, possibly through G protein-coupled receptor signaling, molecular transduction, and nucleosome DNA-binding pathways. Furthermore, ac4C exhibits critical physiological functions in male reproduction, with its abundance dynamically fluctuating during spermatogenesis (Chen et al., 2022). Concurrently, male mice lacking NAT10 display a complete absence of mature sperm in the epididymis and a marked reduction in germ cell numbers within the seminiferous tubules. This indicates that ac4C is essential for testicular development and male fertility.

#### 3.2.3 ac4C responds to cellular stress

In addition to regulating development, ac4C modification also contributes to the adaptive responses of cells under stress, helping them cope with fluctuations in their internal and external environments. In the absence of tRNA ac4C, decreased tRNALeu levels cause ribosomes to stall and collide at the corresponding codon, activating the GCN2-eIF2α-mediated integrated stress response, which inhibits cell growth (Thalalla Gamage et al., 2025). Furthermore, ac4C levels in yeast mRNA rise noticeably under oxidative stress (Tardu et al., 2019), pointing to a dynamic role for this modification in stress adaptation.

## 4 The NAT10/ac4C axis in multi-system diseases

Given the ongoing controversies over ac4C detection methods, the findings discussed below should be viewed as exploratory evidence unless they have been validated by orthogonal experiments. This review classifies ac4C target genes into two categories: orthogonally verified sites and candidate sites. For details, see Supplementary Table S2. The evidence was assessed along two independent dimensions. The first dimension reflects clinical relevance, which depends on the biological model used. These models include human tissue samples, such as transcriptomic data from clinical specimens, tissue microarrays, or public databases, as well as animal models (*in vivo*) and cellular models (*in vitro*). Human tissue samples indicate direct clinical relevance. Animal models suggest that *in vivo* function has been preliminarily confirmed, but human evidence is still needed for clinical translation. Cellular experiments require further confirmation in higher-level models. The second dimension reflects the depth of mechanism validation, which depends on the rigor of experimental validation. This includes whether the ac4C site was confirmed by different methods, such as dual-luciferase reporter assays, site-directed mutagenesis, and whether functional consequences were established through genetic rescue experiments. These two dimensions are presented separately and are not merged into a single hierarchy (Figure 3).

**FIGURE 3:**
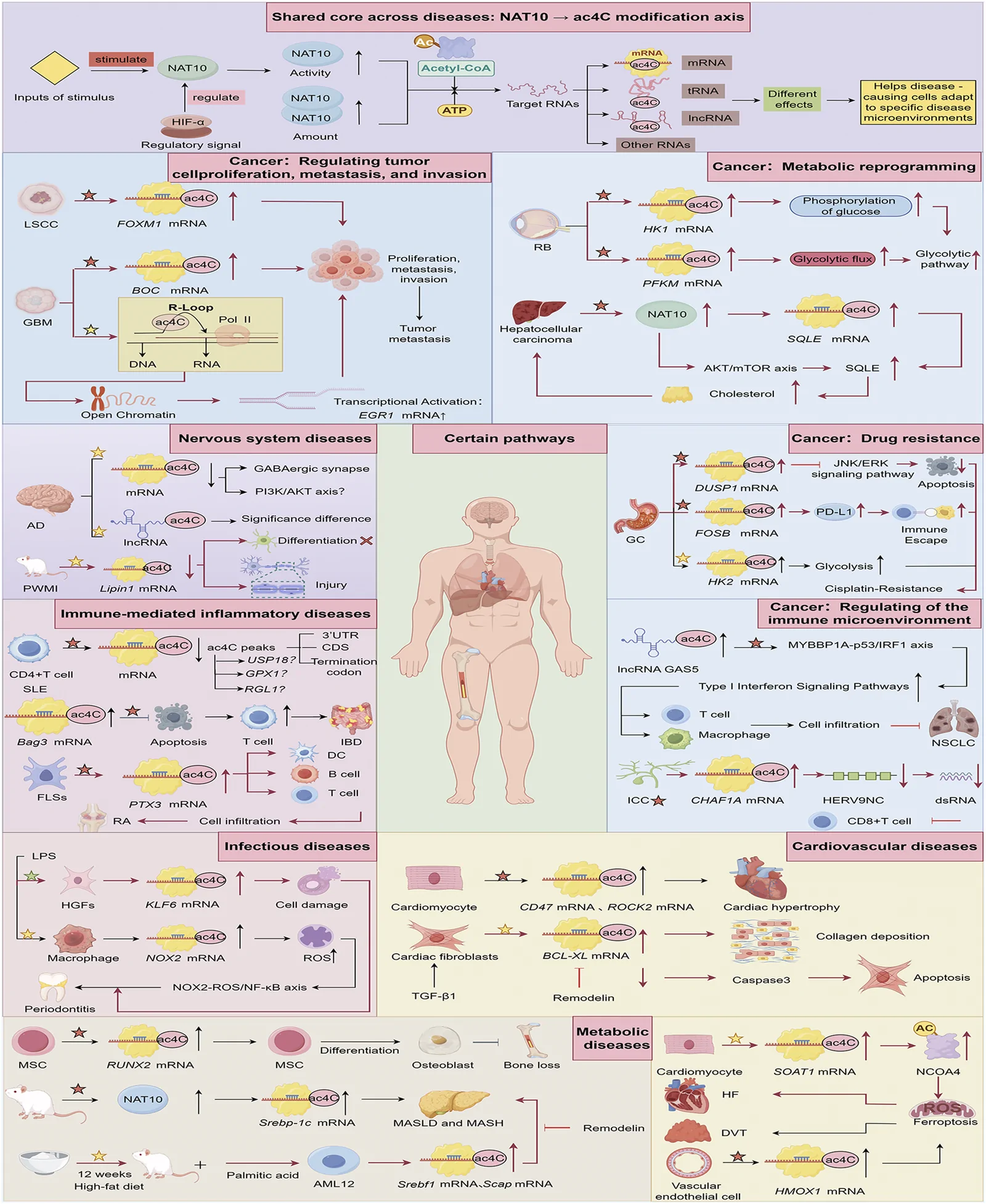
The NAT10/ac4C axis in human diseases. A schematic overview of how dysregulated ac4C modification contributes to the pathogenesis of cancer, cardiovascular diseases, metabolic disorders, infectious diseases, immune-mediated inflammatory conditions, and nervous system diseases. Pathways are shown with their highest biological model used: human tissue support (red★), animal model support without human data (yellow★), or cell-based evidence only (green★). Red pathways indicate orthogonal experimental validation; black pathways indicate no orthogonal validation.

### 4.1 Cancer

The NAT10/ac4C axis emerges as a driver of tumor malignancy by regulating critical oncogenic transcripts. These transcripts influence processes ranging from uncontrolled proliferation to drug resistance. The axis operates through four main areas: proliferation and metastasis, metabolic reprogramming, drug resistance, and immune microenvironment remodeling. Each area is discussed below. It should be noted that ac4C detection methods remain controversial. Scientists disagree on whether ac4C is truly present on mRNA. NAT10 also possesses ac4C-independent functions that may contribute to the observed phenotypes. Therefore, unless orthogonal methods are used to confirm the findings, the ac4C sites mentioned below require further validation. The evidence in this section comes mainly from human tissue, cancer cell lines, and mouse models.

#### 4.1.1 Regulating tumor cell proliferation, metastasis, and invasion

The NAT10/ac4C axis contributes to tumor cell proliferation, metastasis, and invasion through at least two ways. The first, more commonly reported, acts post-transcriptionally by stabilizing mRNA. In glioblastoma (GBM), ac4C modification of *BOC* mRNA has been reported to increase its stability and translation efficiency, promoting stem cell migration and proliferation (Lin et al., 2026). A similar stabilization-associated phenotype has been noted in laryngeal squamous cell carcinoma (LSCC), though orthogonal validation is still needed (Li et al., 2023). The second route, so far found only in GBM, operates at the transcriptional level. Here, NAT10 has been shown to bind R-loops and deposit ac4C onto R-loop RNAs. This may stabilize promoter-associated R-loops, keep local chromatin open, and activate EGR1 transcription, thus promoting stem cell self-renewal and tumor initiation (Wu X. et al., 2026). That both routes operate in GBM speaks to NAT10’s versatility, yet the R-loop route has not been seen in other cancers. In GBM, NAT10 expression is driven by distinct upstream signals: in most tumor cells, hypoxia induces HIF1α, which activates its transcription; in glioma stem cells, OLIG1 upregulates NAT10’s expression to maintain stemness. What remains unclear is the relative contribution of each pathway to the tumor and whether this balance shifts during the progression of GBM.

#### 4.1.2 Metabolic reprogramming

NAT10/ac4C-driven glycolysis is a key part of metabolic reprogramming in cancer. In retinoblastoma (RB), this axis has been reported to target two glycolytic enzymes: *HK1*, which catalyzes the initiating step of glucose phosphorylation (Xu et al., 2025), and *PFKFB3*, a rate-limiting enzyme that controls glycolytic flux (Li Z. et al., 2026). ac4C modification of both transcripts appears to enhance mRNA stability, reinforcing the Warburg effect. However, the two enzymes are not modified equally: when NAT10 is overexpressed, ac4C levels on *PFKFB3* mRNA are much higher than those on *HK1* mRNA, suggesting that *PFKFB3* may be a preferred target. This differential modification suggests a target hierarchy in which selectivity depends on cellular context and NAT10 levels. Beyond glycolysis, the NAT10/ac4C axis also participates in other metabolic processes, including cholesterol metabolism (Wang et al., 2025a), helping cancer cells survive in low-oxygen conditions and compete for limited nutrients.

#### 4.1.3 Drug resistance

Treatment resistance is now viewed as a multidimensional problem. Apoptotic bypass, immune escape, and metabolic adaptation are coupled through shared regulatory nodes. In gastric cancer (GC), the NAT10/ac4C axis seems to couple these processes so as to drive cisplatin resistance. ac4C-mediated upregulation of *DUSP1* may suppress JNK/ERK signaling, reducing cisplatin-induced apoptosis; in parallel, ac4C appears to stabilize *FOSB* mRNA, upregulating PD-L1 and facilitating immune evasion (Qian et al., 2026). GNE-493, a NAT10 inhibitor, has been shown to lower ac4C levels on *HK2* mRNA and reverse cisplatin resistance, bringing a metabolic side into this picture (Wang T. et al., 2026). By stabilizing various downstream transcripts, the NAT10/ac4C axis may regulate responses to chemotherapy, targeted therapy, and immunotherapy, making it a promising target for overcoming resistance across multiple treatment types. The relative contributions of these coupled mechanisms, however, are likely to vary in tumor types and treatment contexts.

#### 4.1.4 Regulation of the immune microenvironment

Depending on the cell type and which transcripts are modified, the NAT10/ac4C axis exerts bidirectional control over the tumor immune microenvironment. In non-small cell lung cancer (NSCLC), ac4C-mediated stabilization of the lncRNA GAS5 has been reported to activate the MYBBP1A–p53/IRF1 axis, which enhances macrophage and T-cell infiltration (Wang Z. et al., 2024). Conversely, upregulation of the NAT10/ac4C/*CHAF1A* axis in intrahepatic cholangiocarcinoma (ICC) could suppress the expression of the nuclear transposon *HERV9NC*, reduce dsRNA accumulation, and weaken the innate immune response. This led to reduced CD8^+^ T-cell infiltration and created an immunosuppressive tumor microenvironment (Lu et al., 2026). These contrasting outcomes underscore a broader principle: the NAT10/ac4C axis in immune responses does more than stabilize mRNAs. It also acts through lncRNAs and chromatin-level control of repetitive elements. This versatility means that targeting the axis may need to be tailored to each tumor’s immune landscape.

In cancer, the NAT10/ac4C axis acts as a central hub for stress response. Under conditions such as hypoxia and chemotherapy exposure, this axis enhances pathways already active in target cells, conferring an adaptive advantage on tumor cells. The specific dimension enhanced depends on the cells’ intrinsic transcriptome. For example, tumors such as RB that rely on glycolysis preferentially reprogram metabolic pathways, while ICC uses this axis to strengthen its inherent immunosuppressive microenvironment. Substrate selectivity adds another layer to this tumor-type specificity: even within the same pathway, NAT10 targets are not all modified equally. In glioma stem cells, the NAT10/ac4C axis can also activate previously silent genes through R-loop-mediated chromatin remodeling, suggesting its function goes beyond reinforcing existing pathways. However, whether this mechanism is unique to GBM or reflects technical limitations in studying chromatin regulation in other tumors remains unclear. Finally, a key issue runs through the entire field: most studies attribute the oncogenic phenotype to mRNA acetylation but fail to distinguish the contributions of tRNA, rRNA, or the non-catalytic function of NAT10 in experiments.

The NAT10/ac4C axis has been studied most extensively in oncology. Most studies include clinical samples, cell lines, animal models, perturbation experiments, and orthogonal validation, providing strong evidence. Some studies have established a complete causal chain from ac4C modification to functional phenotypes using multi-omics and orthogonal validation. However, coverage varies across cancer types. Most work focuses on hepatocellular carcinoma, gastric cancer, and colorectal cancer, and some studies lack ac4C site-specific validation.

### 4.2 Cardiovascular disease

In the cardiovascular system, the NAT10/ac4C axis is implicated in two distinct pathological processes: structural remodeling and cell death. Its expression correlates with disease progression and could aid in disease monitoring. Pharmacological or genetic inhibition of the axis reduces cardiac remodeling and thrombus burden, suggesting this pathway as a therapeutic target. However, there are a few cautions to consider. NAT10 has both acetyltransferase activity and ac4C-independent roles, including nucleolar maintenance and ribosome assembly. Pathological states induced by NAT10 disruption may involve a combination of these two functions. Since ac4C readers and erasers remain unidentified, the full regulatory network is still unclear. Clinical translation will require further work.

In heart failure, the NAT10/ac4C axis has been reported to stabilize mRNAs of pro-remodeling genes like *CD47* and *ROCK2*, driving cardiomyocyte hypertrophy and myofibroblast activation, although orthogonal validation of these sites is still lacking (Shi et al., 2023). In myocardial infarction models and TGF-β1-stimulated cardiac fibroblasts, ac4C has been shown to drive fibroblast proliferation and collagen synthesis, thereby promoting myocardial fibrosis (Li et al., 2024). On the other hand, the researchers found that Remodelin downregulated the NAT10/ac4C/BCL-XL signaling pathway, activated Caspase-3, and triggered apoptosis in cardiac fibroblasts. Regarding cell death, ac4C may exacerbate heart failure (HF) by stabilizing *SOAT1* mRNA, which promotes NCOA4-dependent ferritinophagy and ferroptosis (Deng et al., 2025). Similarly, it is associated with deep vein thrombosis (DVT) by stabilizing *HMOX1* mRNA, leading to iron accumulation and endothelial dysfunction (Zhang et al., 2026). Beyond these mechanisms, ac4C displays notable tissue-specific effects: ac4C appears to promote ferroptosis in cardiovascular cells, yet may suppress ferroptosis in colorectal cancer (Zheng et al., 2022). Furthermore, ac4C may exert bidirectional effects in heart failure, potentially promoting adaptive remodeling in early stages but accelerating deterioration in advanced stages. This shift likely depends on disease progression and cellular context, suggesting that therapy should be tailored rather than broadly inhibiting the axis.

The strength of evidence from existing studies can be assessed along two dimensions: clinical relevance and depth of mechanism validation. In terms of clinical relevance, the DVT study by Zhang et al. and the myocardial hypertrophy study by Shi et al. both utilized human tissue samples, animal models, and cellular experiments, demonstrating the highest potential for clinical translation. By contrast, the heart failure study by Deng et al. and the myocardial fibrosis study by Li et al. are based solely on animal and cellular models. In terms of the depth of mechanism validation, the DVT study established a complete causal chain linking ac4C to the disease through experiments such as knockdown, overexpression, conditional knockout, pharmacological inhibition, and *HMOX1* rescue. Shi et al. used SLAM-seq and Ribo-seq to validate their targets at both RNA stability and translation levels, though orthogonal confirmation is still lacking. Research on heart failure has provided evidence for the ferroptosis mechanism through *NCOA4* rescue experiments and dual-luciferase reporter assays, but lacks a genetic knockout of NAT10. Although the target genes in the myocardial fibrosis study have been validated by dual-luciferase assays, NAT10 disruption relies solely on Remodelin treatment and lacks genetic intervention and rescue experiments, resulting in a relatively weak chain of evidence.

### 4.3 Metabolic disorders

In metabolic diseases, the NAT10/ac4C axis correlates with hepatic lipid accumulation and bone density regulation. Preclinical evidence from animal models suggests that targeting this axis improves lipid deposition and attenuates bone loss, pointing to its potential as a marker. Still, a few caveats are worth noting. Some studies lack orthogonal validation of ac4C targets; the readers and erasers of ac4C have not been identified; and whether NAT10’s housekeeping functions contribute to the observed phenotypes remains unclear. Further validation is therefore required to confirm whether these metabolic effects are genuinely mediated by the NAT10/ac4C axis. The evidence discussed in this section derives primarily from hepatocytes, bone marrow-derived mesenchymal stem cells (BMSCs), and mouse models.

In osteoporosis, the downregulation of the NAT10/ac4C axis is implicated in RUNX2-mediated osteogenic differentiation. Yang et al. (2021) observed that ac4C may upregulate *Runx2*, promoting the differentiation of BMSCs into osteoblasts and slowing bone loss, although orthogonal validation is still lacking. ac4C appears to stabilize mRNAs encoding lipid metabolism genes as well. In metabolic dysfunction-associated steatotic liver disease (MASLD) and metabolic dysfunction-associated steatohepatitis (MASH), ac4C was elevated on hepatic *Srebp-1c* and on other lipogenic genes including *Fasn*, *Acc1*, and *Scd1*, while NAT10 knockout reduced fatty acid β-oxidation genes such as *Mcad, Acox1, Ppara,* and *Cpt1a*, though orthogonal validation is still needed (Yang et al., 2024). Wang Z. et al. (2024) further demonstrated that in the livers of mice fed a high-fat diet and in AML12 cells treated with palmitic acid, ac4C levels on *Srebf1* and its molecular chaperone *Scap* were significantly increased, accompanied by increased expression of lipogenic enzymes. This raises the possibility that chaperone transcripts themselves may serve as NAT10 targets. Notably, the NAT10/ac4C axis acts as a pathogenic driver in MASLD but as a protective factor in osteoporosis: the same pathway shows opposing effects within the same disease category. This contrast highlights that NAT10-targeted therapies must account for tissue specificity, as systemic inhibition may improve MASLD while worsening bone loss.

The same two dimensions apply to metabolic studies. Clinically, Yang Y. et al. used human tissue samples, animal models, and cellular experiments in their MASLD study, offering the highest potential for clinical translation. The bone metabolism study similarly included human tissue, animal models, and cellular models. By contrast, Wang et al. relied on animal and cellular models without human tissue validation. Regarding depth of mechanism validation, Yang Y. et al. integrated acRIP-seq and RNA-seq to establish a comprehensive line of evidence linking ac4C modification to lipid metabolism phenotypes. Wang et al. employed dual-luciferase reporter assays to validate ac4C sites on *Srebf1* and *Scap*, providing mechanistic depth. The bone metabolism study by Yang W. et al. included acRIP-seq, siRNA-mediated knockdown and overexpression, and *RUNX2* rescue experiments, yet lacks *in vivo* validation to confirm RUNX2 as the direct mediator of the observed phenotype. Except for Wang et al., who used dual-luciferase assays, ac4C sites in the other studies rely on acRIP-seq and await orthogonal confirmation.

### 4.4 Infectious diseases

In infectious diseases, the NAT10/ac4C axis correlates with inflammatory cytokine release and tissue injury, suggesting its potential as a marker of infection-associated inflammation and a therapeutic target. Mechanistically, it may modulate inflammation-related gene expression by influencing the stability and translation of key mRNAs. However, most evidence comes from NAT10 perturbation experiments. These studies do not distinguish ac4C-dependent from ac4C-independent effects. Furthermore, since the ac4C readers and erasers have not yet been identified, the dynamic regulation of ac4C in immune cells remains unclear. Therefore, the specific role of the NAT10/ac4C axis in infectious diseases should be interpreted with caution. And its clinical translation requires further validation. Evidence discussed in this section derives primarily from cellular and animal models.

In lipopolysaccharide (LPS)-induced human gingival fibroblasts (HGFs), Liao et al. (2024) found that an abnormal elevation of ac4C on *KLF6* mRNA may mediate periodontitis and cellular damage; inhibition of NAT10 could protect HGFs. Zhang et al. (2023) further observed in macrophages that knocking down NAT10 can reduce NOX2 expression and ROS production, and suppress the NF-κB signaling pathway. Remodelin reduced macrophage infiltration and alleviated alveolar bone resorption. However, this study did not directly detect ac4C modification of *NOX2* mRNA. So *NOX2* should be regarded as a downstream effector molecule regulated by NAT10, and its status as an ac4C target gene requires further experimental validation. Although the two studies differ in cell type and downstream targets, they both support a pro-inflammatory role for the NAT10/ac4C axis in periodontitis.

In terms of clinical relevance, Liao et al. relied solely on cellular experiments, without animal or human tissue validation. Zhang et al. included both cell and animal models, but human tissue data are still lacking. Mechanistically, Liao et al. used acRIP-qPCR, dual-luciferase assays, and *KLF6* rescue experiments to confirm that NAT10 regulates inflammation by ac4C-modifying *KLF6* mRNA; Zhang et al. used RNA-seq and Remodelin treatment to confirm the pro-inflammatory role of NAT10. Only *KLF6* has been validated by orthogonal approaches, while the remaining findings require further confirmation.

### 4.5 Immune-mediated inflammatorydiseases

Immune-mediated inflammatory diseases are driven by disrupted immune homeostasis and sustained effector cell activation. These cells rely on stable survival signals to stay active. The NAT10/ac4C axis appears to reinforce the survival support. Experiments indicate that targeting NAT10 can reduce tissue inflammation and immune cell infiltration, supporting it as a potential intervention target. However, NAT10 has both ac4C-dependent and ac4C-independent functions, and their effects are hard to separate. In addition, the ac4C regulatory network remains incomplete, which limits our understanding of the axis in immune-mediated inflammation.

The NAT10/ac4C axis seems to act differently in immune-mediated inflammatory diseases, depending on the disease and cell type. *PTX3* mRNA, when heavily modified with ac4C, appears to gain stability and translation efficiency in fibroblast-like synoviocytes (FLS) and the synovium of rheumatoid arthritis (RA) patients. This promotes FLS migration and infiltration, while also increasing infiltration by dendritic cells, B cells, and T cells, thereby exacerbating arthritis (Liu D. et al., 2024). In inflammatory bowel disease (IBD), an *in vivo* transfer model directly demonstrated that T cells lacking NAT10 lose their ability to induce colitis. ac4C may maintain *Bag3* mRNA stability and inhibit T cells' apoptosis, allowing T cells to survive and drive intestinal inflammation. However, orthogonal validation is still lacking (Li H. et al., 2025). The former enhances effector cell function, while the latter supports their survival. In SLE, ac4C modifications in CD4^+^ T cells are primarily concentrated in the CDS, 3' UTR, and stop codon regions, a distribution that differs from that previously reported for HeLa cells. This may stem from variations in sample size, type, or disease subtype. Furthermore, the role of the NAT10/ac4C axis in SLE remains unclear. *USP18*, *GPX1*, and *RGL1* may be its downstream targets (Guo et al., 2020). It is worth noting that NAT10 expression is reduced in SLE patients, contrary to the pattern observed in RA and IBD. This difference may reflect cell types or the pathophysiological mechanisms of SLE.

The same two dimensions apply to immune-mediated inflammatory diseases. Liu et al. (RA) and Li et al. (IBD) used human samples, animal models, and cells, giving their findings the highest translational potential. In contrast, Guo et al. (SLE) relied only on human samples, without animal or cellular validation. Mechanistically, the RA evidence is the most complete, spanning acRIP-seq, luciferase reporter validation, NAT10 knockdown/overexpression/haploinsufficiency, and Remodelin treatment. The IBD study offers unique *in vivo* evidence for T cells' pathogenicity. The SLE study, however, remains at the associative level, based on acRIP-seq and LC-MS/MS data from patient CD4^+^ T cells. These gaps highlight the need for causal studies to clarify the role of the NAT10/ac4C axis in immune-mediated inflammatory diseases.

### 4.6 Nervous system diseases

Nervous system diseases are characterized by diverse pathological mechanisms, including neurodegeneration and synaptic dysfunction, with limited options for early diagnosis. ac4C maintains neuronal health and synaptic function by stabilizing mRNA and enhancing translation. Abnormal ac4C modification may contribute to synaptic protein loss and impaired myelination, pointing to the NAT10/ac4C axis as a potential target in nervous system disease. However, evidence in this field is limited, and existing findings lack functional validation and causal evidence. Furthermore, the regulatory network controlling ac4C homeostasis remains poorly defined, limiting our understanding of neuronal homeostasis and synaptic plasticity. Overall, this field is still in the early exploratory stage. The evidence discussed in this section is primarily derived from mouse models.

In early Alzheimer’s disease (AD), proteins associated with synaptic function decreased, and the ac4C levels of corresponding mRNAs were downregulated (Ji et al., 2025). mRNAs with altered ac4C levels were mainly enriched in GABAergic synapses and the PI3K/AKT signaling pathway (Ma et al., 2023). This suggests that ac4C may drive early synaptic dysfunction in AD by affecting synaptic protein expression. Meanwhile, Ma et al. (2022) conducted a comprehensive analysis using acRIP-seq and RNA-seq, revealing that the abundance of the lncRNA ac4C exhibits marked differences in AD. This finding implicates the NAT10/ac4C axis in AD pathogenesis and progression. However, none of these studies have established specific ac4C-related signaling networks in AD, and all require orthogonal validation. Beyond AD, downregulation of the NAT10/ac4C/*Lipin1* signaling axis in the preterm white matter injury (PWMI) mouse model results in aberrant myelin development and impaired oligodendrocyte precursor cell differentiation (Li X. et al., 2025). In summary, targeting and reversing abnormal acetylation modifications may represent novel strategies for delaying the progression of neurological disorders.

Nervous system diseases represent the area with the weakest evidence in the NAT10/ac4C studies. The same two dimensions apply to this field. Four studies rely on animal models, with only the PWMI study by Li et al. also using cellular experiments. None includes human tissue. The depth of mechanistic validation varies considerably across these studies. In AD research, Ji et al. used acRIP-seq, RNA-seq, and proteomics; the other two AD studies were confined to sequencing and bioinformatics predictions. Target genes identified in different AD models are not directly comparable, as mRNA and lncRNA functions differ. Across all three studies, the evidence remains descriptive, with no causal validation. In contrast, the PWMI study established complete causal chains through point mutations, *in vivo* and *in vitro* perturbations, and other methods. This discrepancy comes from the study design. The PWMI study provides mechanistic validation, whereas the AD studies remain largely descriptive. Overall, there is still a great deal of work to be done in this field to bridge the gap between “describing ac4C changes” and “proving ac4C pathogenicity.”

### 4.7 Cross-system pathological mechanisms

From a cross-disease perspective, the NAT10/ac4C axis operates as a regulatory cascade. Upstream stress signals (hypoxia, inflammation, metabolic imbalance, etc.) activate NAT10, driving ac4C deposition and then altering the fate of target transcripts. This produces downstream effects such as metabolic reprogramming and ultimately contributes to the disease. Physiological and pathological states share the same regulatory cascade. The distinction lies in the nature and duration of the upstream signals. Under physiological conditions, transient, reversible stress signals enable cells to maintain homeostasis; under pathological conditions, sustained, high-intensity stress drives disease processes such as proliferation, immune evasion, and lipid deposition. Within this framework, the NAT10/ac4C axis can be seen as a central adaptive regulatory node that translates environmental stress signals into adaptive programs (Bo et al., 2026). In this way, the axis supports normal cell homeostasis; in pathological settings, it helps pathogenic cells survive and undergo malignant progression.

However, the output of this pathway is subject to multiple regulatory mechanisms. We propose that tissue specificity may determine the direction of NAT10/ac4C output, which varies across cell types and tissues. Cell-intrinsic RNA defines the scope of NAT10’s targets, largely dictating disease progression. In addition, the subcellular localization of NAT10 differs among cell types. In normal cells, NAT10 is primarily localized to the nucleolus; however, in cancer cells, it can translocate to the nucleoplasm, cytoplasm, and even the cell membrane, thereby changing the substrates it can access (Han et al., 2025). Context dependence may regulate pathway switching. In the same cell type under different conditions, the functional output of the NAT10/ac4C axis can shift from maintaining homeostasis to driving pathology. A recent study has shown that in normal hematopoietic stem/progenitor cells (HSPCs), ac4C may enhance the translational efficiency of the key hematopoietic transcription factor *Nfix*, maintaining the self-renewal of hematopoietic stem cells and the differentiation of megakaryocytic and erythroid progenitor cells (Huang et al., 2026). However, in acute myeloid leukemia, ac4C has been reported to enhance translation of the serine transporter *SLC1A4* for serine uptake and the oncogenic transcriptional regulators *HOXA9/MENIN* for serine synthesis, thereby driving leukemogenesis (Zhang S. et al., 2024). This suggests that NAT10 can target different mRNAs and exert different functions depending on cellular state and microenvironmental signals. Building on this, the output of the NAT10/ac4C axis is further shaped by positive feedback loops that lock in its activation and amplify the pathological effects. In GC, HIF-1α/NAT10/*SEPT9*/HIF-1α drives glycolytic addiction (Yang et al., 2023); in myocardial ischemia-reperfusion injury, p53/NAT10/*Mybbp1a*/p53 drives ferroptosis (Qu et al., 2024). Both are positive feedback loops that sustain pathological signaling.

Together, these factors shape the final pathological phenotype. Overall, the NAT10/ac4C axis presents both therapeutic potential and challenges for targeted intervention: inhibiting this axis may be protective in certain tissues or at certain disease stages, but could be counterproductive in others.

## 5 Therapeutic developments and translational barriers

In recent years, NAT10-targeting strategies have expanded beyond Remodelin (a single small-molecule inhibitor) to include PROTAC degraders and repurposed drugs (Supplementary Table S3). Although NAT10 has shown preclinical promise as a therapeutic target, clinical translation still faces multiple barriers. This section outlines the therapeutic potential and risks of NAT10 inhibitors, aiming to provide a critical perspective on future treatments.

### 5.1 Therapeutic potential and stratification strategies

NAT10 is highly expressed in various tumors. Changes in its subcellular localization (from the nucleolus/nucleus to the cytoplasm or cell membrane) are associated with increased invasiveness, suggesting a poor prognosis. And its downstream molecules can be detected, demonstrating potential for monitoring. In summary, this axis may serve as a biomarker reflecting tumor epigenetic transcriptional activity, with potential utility in diagnosis, risk stratification, treatment prediction, and prognosis (Xiong et al., 2026). It is evident that strategies targeting NAT10 hold significant therapeutic potential, but they must be tailored to enzyme characteristics, tumor type, and microenvironment. A comprehensive evaluation should consider several dimensions: ① NAT10 expression levels may determine the target population. Patients with high NAT10 expression may benefit more from NAT10 inhibitors, while those with low NAT10 expression may require alternative treatment strategies. ② The type of downstream pathway may determine the direction of combination therapy. Metabolic pathways may be targeted with glycolysis inhibitors (e.g., 2-DG), while immunosuppressive pathways may be targeted with immune checkpoint blockade (e.g., anti-PD-1/PD-L1 antibodies). ③ Interactions between different pathways may determine the sequence of treatment. Studies have shown that high concentrations of lactate can suppress cytotoxic T cells and natural killer cells and impair antigen presentation (Wang X. et al., 2026). Therefore, tumors with high NAT10 expression, characterized by glycolytic enrichment, are often associated with an immunosuppressive microenvironment. In such cases, a combination of NAT10 inhibitors and metabolic intervention, followed by immunotherapy, may be a priority. However, the clinical feasibility of this approach requires validation through clinical trials.

### 5.2 Limitations of Remodelin

Remodelin is currently the most widely used NAT10 inhibitor, but it has significant limitations. First, its pharmacokinetic and pharmacodynamic parameters (e.g., bioavailability, tissue distribution, and tumor penetration) have not been fully established. Clinical efficacy and safety data are lacking. Second, Remodelin is not a specific inhibitor of NAT10. A study indicates that it is a “cryptic assay interference chemotype” with the potential to interact with multiple protein targets. Some of its effects may involve mechanisms independent of NAT10 (Shrimp et al., 2021). Furthermore, NAT10 is broadly expressed in human tissues and participates in diverse physiological processes, including ribosome biogenesis, nucleolar structure maintenance, and mitosis. Systemic inhibition could cause toxicity in vital organs such as the heart, nervous system, and reproductive organs. Therefore, treatment with Remodelin should be carefully considered.

### 5.3 Tissue-specific targeting strategies

The housekeeping functions of NAT10 in normal tissues suggest that the therapeutic window for systemic NAT10 inhibition is inherently narrow. Strategies targeting NAT10 that lack tissue specificity will inevitably damage normal proliferating or highly metabolically active tissues such as germ cells and neural tissue. To reduce the risk of off-target effects, tissue-specific targeting strategies are important areas for optimization. In recent years, the Sono@NAT10 nanorobot, which releases siRNA upon ultrasound stimulation to achieve specific silencing in the tumor microenvironment (Zhu J. et al., 2025), and the PEI/PC7A/siNAT10 nanoparticles, which target and degrade NAT10 in tumor cells (Liu W. et al., 2025), have been developed. When combined with anti-PD-1 therapy, both approaches demonstrated synergistic antitumor efficacy. These advances suggest that NAT10-targeted strategies are evolving from broad-spectrum inhibitors toward tissue-specific, precision delivery.

### 5.4 Consequences of long-term NAT10 inhibition

To date, the consequences of long-term use of NAT10 inhibitors have not been systematically evaluated. While long-term treatment may inhibit tumor growth, it may also exert stress on tumor cells, leading to the expansion of drug-resistant subpopulations or the activation of compensatory pathways (Li F. et al., 2026). More importantly, NAT10 plays an irreplaceable role in meiosis during spermatogenesis, and its potential function in nervous system development has also been suggested. This highlights that treating NAT10 inhibition as a long-term intervention strategy requires careful consideration.

## 6 Unresolved core field controversies and emerging research frontiers

### 6.1 Unresolved core field controversies

There are several key controversies in the current field of ac4C modification research, involving core issues such as the distribution of the modification, its regulatory mechanisms, and the reliability of detection. Each is discussed below.

#### 6.1.1 The authenticity of ac4C on mRNA

The authenticity of ac4C on mRNA remains one of the most contentious issues in the field. Using a chemical reduction-based approach (RedaC:T-seq), Arango et al. reported widespread ac4C deposition in human mRNA (Arango et al., 2022). Reanalyzing this dataset, Georgeson and Schwartz challenged these findings, pointing out that the C→T mismatches were almost exclusively concentrated in a single replicate; the library complexity was extremely low, with the majority of reads derived from repeated sequencing of a small number of source molecules; after removing these duplicate reads, the abnormal pattern largely disappeared, and all types of mismatches increased uniformly rather than being limited to C→T. This is inconsistent with the chemical signature of ac4C, suggesting that the initial observations likely arose from amplification bias (Georgeson and Schwartz, 2024). In response, Beiki et al. reaffirmed that the C→T mismatch signals showed consistency across two replicates, identifying approximately 2,000 sites and supporting the existence of mRNA ac4C (Beiki et al., 2024). The core issue is distinguishing real signals from background noise in low-abundance modifications detected by chemical methods. True signals could easily be masked by sequencing errors inherent to the platform and random mutations introduced during PCR amplification. Achour and Oberdoerffer noted that, given NAT10’s involvement in ribosome biogenesis, nucleolar maintenance, and other processes, isolating the effects of ac4C on specific mRNAs from other NAT10 activities remains difficult (Achour and Oberdoerffer, 2024). Extending this reasoning, any phenotypic outcomes observed in NAT10 perturbation experiments could be attributed to the loss of ac4C, impairment of NAT10’s ac4C-independent functions, or a combination of both. Current experimental approaches cannot disentangle these three possibilities. In summary, the actual existence and biological significance of mRNA ac4C remain open questions. No current method gives a definitive answer. Antibody-based approaches lack specificity. Chemical methods suffer from poor signal-to-noise ratios. Genetic manipulations cannot separate ac4C effects from NAT10’s other functions. Until better tools become available, reported ac4C site maps should be treated with caution.

#### 6.1.2 Unresolved readers and erasers of ac4C modification

Ac4C is dynamically regulated by reader, writer, and eraser proteins. As discussed in previous sections, the only currently known ac4C writer is NAT10. The other two proteins have not yet been sufficiently validated. SIRT7 can deacetylate ac4C on rRNA *in vitro* (Xu et al., 2021), but whether it performs a similar function on mRNA remains inconclusive. Regarding readers, research has proposed several candidate proteins. NOP58 was an early ac4C-binding protein identified through *in vitro* RNA pull-down assays; subsequent studies found that it colocalizes with ac4C-modified mRNA in stress granules (Kudrin et al., 2024). In certain tumors, EEF2 (Liu H. et al., 2024) and MYBBP1A (Wang H. et al., 2026) have also been reported to bind to ac4C. Currently, there are no universally recognized readers. Overall, how ac4C is recognized and removed is unresolved and requires future studies.

#### 6.1.3 Technical inconsistency across detection methods

Different methods used to investigate the same biological question may yield conflicting conclusions. This may be due to differences in the focus of ac4C detection among the methods, or it may result from technical biases in the methods themselves. Currently, the field of ac4C lacks systematic cross-method comparisons, and the consistency of results across different technologies remains unclear. Future large-scale, systematic evaluations are needed to establish universal reference standards for ac4C detection.

#### 6.1.4 Non-catalytic functions of NAT10

Beyond depositing ac4C, NAT10 can also act through mechanisms that do not depend on its catalytic activity. While these non-catalytic functions have been demonstrated, their relative contributions across different tissues and cellular states are uncertain. Taking normal hematopoietic stem cells as an example, NAT10 maintains self-renewal through two mechanisms: first, as an acetyltransferase, it appears to enhance the translation of transcription factors such as *Nfix* via ac4C modification (Huang et al., 2026). Second, as an RNA-binding protein, it has been reported to protect transcripts from degradation by recognizing motifs in the 3′UTR of mRNAs. This process appears to be independent of ac4C (Li W. et al., 2026). Both mechanisms have been observed in the same cell type, suggesting that NAT10 may adjust the balance between its catalytic and non-catalytic functions depending on the cellular state. However, the factors determining this balance have yet to be elucidated. Do these non-catalytic functions independently contribute to physiological or pathological processes? How do the contributions of catalytic and non-catalytic functions dynamically change in different disease stages? Which pathological conditions primarily rely on NAT10’s non-catalytic functions rather than its modification activity? These questions remain open. It is unclear whether the phenotypes observed after NAT10 knockout come from loss of ac4C or loss of its non-enzymatic functions.

### 6.2 Emerging research frontiers

Despite significant progress, numerous challenges persist in translating our understanding of ac4C into clinical benefits. Based on this review, we propose several focus areas for future research.

Basic research frontiers:

1. Single-cell and spatial ac4C mapping: Most data generated by current detection methods represent “averages” across large numbers of cells, which can mask differences between individual cells. Recent studies in melanoma have combined single-cell transcriptomics with machine learning algorithms to construct ac4C-related gene signatures (acRGS), revealing differences in ac4C distribution across cell subsets: ac4C modification signals are enriched in melanocytes (Liu J. et al., 2025). Future research should focus on developing ac4C sequencing methods for low-input samples (e.g., microfluidic chips) to reach single-cell level resolution and enable spatial mapping of ac4C in tissues. This could be achieved, for instance, by integrating spatial transcriptomics with ac4C imaging.
2. Multi-omics combined analysis: The function of ac4C depends on multi-level regulation, including NAT10 expression levels, target transcript abundance, and the coordination of other modifications such as m6A and m5C. Future research should integrate multiple approaches, such as ac4C-seq, RNA-seq, and proteomics, within the same biological sample to establish complete causal chains.
3. Machine learning algorithms for modification prediction: As data accumulates, deep learning models are expected to achieve high-precision ac4C site prediction. Given the current technical bottlenecks, future studies should prioritize orthogonal data, such as ac4C-seq and LC-MS/MS, as training data to improve model specificity and accuracy; develop species-specific training models to address cross-species generalization bottlenecks; and build a comprehensive learning framework that integrates RNA structure prediction with intracellular environmental parameters to improve context-dependent prediction capabilities *in vivo*.
4. Coordination with other RNA modifications: As research has progressed, the relationship between ac4C and other RNA modifications, such as m6A and 5-methylcytidine (m5C), has begun to attract attention. ac4C and m6A interact by acting as upstream and downstream components of the same pathway. ac4C is suggested to stabilize *YTHDC1* mRNA (an m6A-reading protein), and enhance m6A-dependent glycolysis in osteosarcoma (Mei et al., 2024). ac4C and m5C are functionally complementary, working synergistically to establish an immunosuppressive tumor microenvironment. m5C regulates DNA repair, energy metabolism, and cell death checkpoints, enabling cells to adapt to oxidative and energy stress; ac4C coordinates the survival of cancer cells under stress by stabilizing oncogenic transcripts and increasing translational output (Zhong et al., 2026). This network suggests that the NAT10/ac4C axis reflects a broader epitranscriptomic state that cannot be explained by any single modification alone. Does the collaborative mechanism between ac4C, m^6^A, and m^5^C hold true in additional cell types and disease contexts? Are there other pathways that regulate the interactions among these three? Are there potential interactions between ac4C and other modifications, such as m7G? When multiple modifications coexist on the same transcript, what is their combined effect on downstream processes? These questions await future investigation.

Translational frontiers:

1. mRNA therapy (IVT modified mRNA vaccines): Schiffers et al. demonstrated that, in *in vitro* transcribed mRNA, the anti-inflammatory effect of ac4C is comparable to that of m1Ψ. However, because m1Ψ reduces the translation elongation rate by nearly 50%, it readily induces ribosomal collisions and +1 frameshifts, thereby limiting protein yield (Schiffers et al., 2026). This study extends the biochemical characteristics of endogenous ac4C—which accelerates translation—to the therapeutic design of synthetic mRNA. In the future, modification strategies for mRNA therapeutics can choose between ac4C and m1Ψ based on therapeutic goals, or use a combination of both modifications. This opens new avenues for safer and more effective mRNA vaccines and therapeutics.
2. NAT10 selective PROTAC degrader and tissue-specific inhibitor design: Since NAT10 is expressed in both normal and tumor cells, nonspecific inhibition may impair normal cell function. Therefore, the development of highly selective delivery systems is crucial. Nanocarriers such as lipid nanoparticles have demonstrated the potential to improve tumor drug accumulation in other targeted therapies. But their application in NAT10 inhibitors is still in its infancy. Recent studies have shown that the proteolysis-targeting chimera (PROTAC) NP1192 can achieve specific degradation of NAT10, demonstrating higher selectivity (Ao et al., 2025). This marks progress in the shift from “inhibition” to “protein degradation” as an accurate strategy. However, the clinical translation of PROTACs faces significant challenges. Their high molecular weight and complex structure typically result in low water solubility, high lipophilicity, and limited cell membrane permeability, ultimately leading to insufficient oral bioavailability and poor pharmacokinetic performance (Feng et al., 2025). Future research directions may focus on nanoparticle-based PROTAC delivery systems that improve pharmacokinetics and tumor accumulation; and stimulus-responsive PROTAC prodrug strategies that respond to characteristics of the tumor microenvironment, such as low pH and highly expressed enzymes, thereby achieving controlled release.
3. Prospective clinical cohort study design: Currently, NAT10-targeted therapy remains in the preclinical stage, and clinical cohort data are lacking. Nevertheless, the prognostic model ARGSig (ac4C-related gene signature) in lung adenocarcinoma has preliminarily demonstrated its ability to predict patients’ sensitivity to immune checkpoint inhibitors and chemotherapy drugs, providing an important basis for patient stratification (Wang et al., 2025b). Future efforts should focus on conducting multicenter, large-scale prospective cohort studies to systematically evaluate the clinical value of the NAT10/ac4C axis. Building on this foundation, parameters such as NAT10 expression levels, ac4C downstream pathways, and tumor microenvironment characteristics can be further integrated to construct a composite biomarker model, enabling more precise patient selection.

In summary, the future of the NAT10/ac4C axis in clinical translation hinges on a deeper understanding of its fundamental biology. By addressing the critical questions outlined above, and with the continued development of tools to study and manipulate this modification, this axis holds substantial promise as a source of novel biomarkers and therapeutic targets for a wide spectrum of human diseases.

## Glossary

### Core concepts

Epitranscriptomics: The study of all chemical modifications on RNA molecules and their regulatory functions in gene expression
ac4C: N4-acetylcytidine, a chemical modification on RNA catalyzed by NAT10
**m** ^**6**^ **A**: N6-methyladenosine, the most abundant internal modification in eukaryotic mRNA, is frequently used as a reference for comparison with ac4C
**m** ^**5**^ **C**: 5-methylcytidine, a common RNA modification
2′-O-methylation: 2′-O-methylation of the ribose 2′hydroxyl group, which can alter RNA conformation and stability

### Regulatory proteins

NAT10: N-acetyltransferase 10, the only catalytic enzyme of ac4C, has both acetyltransferase activity and RNA-binding ability and is involved in various processes, including ribosome biogenesis, cell cycle regulation, and DNA damage response
GNAT family: GCN5-related N-acetyltransferase family, a large acetyltransferase superfamily to which NAT10 belongs
Writer: An enzyme that catalyzes the deposition of a chemical modification onto RNA
Reader: A protein that specifically recognizes and binds to a modified RNA base
Eraser: An enzyme that removes a chemical modification from RNA

### Technical methods

acRIP-seq: Acetylated RNA immunoprecipitation sequencing, an antibody-based method for transcriptome-wide ac4C detection
ac4C-seq/RedaC:T-seq: Chemical reduction-based ac4C detection methods that exploit selective reduction of ac4C to generate C→T mismatches during reverse transcription, enabling single-base resolution
LC-MS/MS: Liquid chromatography-tandem mass spectrometry, used for absolute quantification of ac4C
Nanopore sequencing: Direct RNA sequencing using nanopores, a single-molecule sequencing technology
Computational prediction: Machine learning/deep learning-based prediction of ac4C sites
Machine learning (ML): A computational approach that enables computers to learn patterns from data and make predictions on unseen data
Single-cell assay: Methods for detecting ac4C at the single-cell level
FMPLA: Fluorine metabolic labeling-mediated proximity ligation assay, a single-cell imaging technique for *in situ* detection of ac4C-modified RNA
Dual luciferase assay: A molecular biology method that quantifies gene expression regulation by measuring firefly and renilla luciferase activities
Point mutation: Substitution of a single nucleotide, commonly used to validate the functional contribution of a modification site
Orthogonal validation: Cross-validation of a conclusion using independent methods based on different principles

### Mechanism-related

LLPS: Liquid-liquid phase separation, a physicochemical process driving the formation of membrane-less organelles
Acetyl-AMP: Acetyl-adenosine monophosphate, an activated intermediate in acetyl group transfer during ac4C modification
