# Supplementary material for: The NAT10/ac4C axis: bridging RNA epigenetics to human diseases
Source: Front Cell Dev Biol. 2026 Oct 28;14:1905147. doi: 10.3389/fcell.2026.1905147 (PMC13752856; doi:10.3389/fcell.2026.1905147)
Supplement: Supplementary file 1 [file Table1.docx]

**Supplementary Table S1:** **Overview of ac4C detection approaches**

| **Method** | **Principle** | **Advantages** | **Limitations** | **Sensitivity/Input** |
| --- | --- | --- | --- | --- |
| **acRIP-seq** | Anti-ac4C antibody enrichment of ac4C-containing RNA fragments followed by high-throughput sequencing | High throughput; suitable for transcriptome-wide screening | Resolution limited to 100–200 nt; cannot pinpoint single-base sites; antibody cross-reactivity generates false positives; enrichment signals are biased by transcript abundance | Microgram-level RNA (ac4C LACE-seq down to 50 oocytes) |
| **ac4C-seq** | NaBH₃CN reduction of ac4C induces C→T mismatches during reverse transcription to map modifications | Single-base resolution; antibody-independent | Reduction efficiency is sensitive to pH and temperature; polymerase termination causes signal loss; secondary structures hinder reagent accessibility | Microgram-level RNA; relatively high input requirement |
| **LC-MS/MS** | Chromatographic separation + mass spectrometry for direct ac4C nucleoside identification | Antibody-independent; absolute quantification | Extremely low abundance in mRNA; rRNA contamination severely interferes; ~25% of the free ac4C nucleoside is deacetylated at pH 7 within 18 h; provides no positional information about ac4C sites | Microgram-level RNA |
| **Nanopore sequencing** | Current signal changes as RNA traverses a nanopore, with modification identification via deep learning models | Single-molecule/single-base resolution; simultaneous detection of multiple modifications | Limited accuracy; poor consistency across tools; low-abundance signals are easily masked | Microgram-level RNA |
| **Computational prediction** | Machine learning/deep learning based on sequence features to predict modification sites | High throughput; low cost | Training data carry acRIP-seq false-positive biases; limited cross-species generalizability; unable to incorporate dynamic information such as secondary structures | Not applicable |
| **FMPLA** | Fluorine metabolic labeling of newly synthesized ac4C + proximity ligation probes + rolling circle amplification → single-cell visualization | Single-cell resolution; enables subcellular localization and dynamic monitoring of ac4C levels | Detects only newly synthesized ac4C (not pre-existing modifications); probe design requires known target sequences; low throughput | Single cells |
