# Supplementary material for: The NAT10/ac4C axis: bridging RNA epigenetics to human diseases
Source: Front Cell Dev Biol. 2026 Oct 28;14:1905147. doi: 10.3389/fcell.2026.1905147 (PMC13752856; doi:10.3389/fcell.2026.1905147)
Supplement: Supplementary file 2 [file Table2.docx]

**Supplementary Table S2:** **Summary of NAT10/ac4C studies**

| **Disease** | **Authors (Year)** | **Targets** | **Proposed effects** | **Validation methods** | **Orthogonal experiments** | **Tissue models** | **Perturbation experiments** | **Treatments** | **Sequencing methods** |
| --- | --- | --- | --- | --- | --- | --- | --- | --- | --- |
| **Osteoporosis** | Yang et al., 2021 | *Runx2* mRNA (candidate site) | ac4C↓→*Runx2* mRNA stability↓ → osteogenic differentiation↓ →bone loss | acRIP-seq, acRIP-qPCR, NAT10 RIP-qPCR, KD, OE, RUNX2 rescue, Remodelin | None | Human, animal, cell | KD, OE | Remodelin | acRIP-seq |
| **MASLD/MASH** | Yang et al., 2024 | *Srebp-1c* mRNA (candidate site) | mRNA stability↑ → lipogenesis↑ → hepatic steatosis | acRIP-seq, RNA-seq, actinomycin D, OE, KD, KO, Cancellation, Remodelin | None | Human, animal, cell | KD, OE, Ad-Cre KO, liver-specific KO | Remodelin | acRIP-seq, RNA-seq |
| **MASLD** | Wang et al., 2024 | *Srebf1/Scap* mRNA (orthogonally verified sites) | mRNA stability↑ → lipogenesis↑ → hepatic steatosis | acRIP-PCR, NAT10 RIP-PCR, dual luciferase, KD, OE, Srebf1 rescue, Remodelin | Dual luciferase | Animal, cell | KD, OE | Remodelin | None |
| **DVT** | Zhang et al., 2026 | *HMOX1* mRNA (candidate site) | mRNA stability↑ → endothelial ferroptosis↑ → DVT | acRIP-seq, acRIP-qPCR, KD, OE, cKO, Remodelin, Fer-1, ZnPP, CoPP, AAV-HMOX1 rescue | None | Human, animal, cell | KD, OE, cKO | Remodelin | acRIP-seq |
| **Cardiac hypertrophy** | Shi et al., 2023 | *CD47/ROCK2* mRNA (candidate sites) | mRNA stability↑ + translation efficiency↑ → hypertrophy/fibrosis | acRIP-seq, SLAM-seq, Ribo-seq, NAT10 KD, OE, Remodelin | None | Human, animal, cell | KD, OE | Remodelin | acRIP-seq |
| **HF** | Deng et al., 2025 | *SOAT1* mRNA (orthogonally verified site) | mRNA stability↑ → NCOA4 acetylation and stabilization↑ → ferritinophagy/ferroptosis↑ → exacerbates HF | acRIP-qPCR, dual luciferase, KD, OE, AAV-shRNA, NCOA4 rescue | Dual luciferase | Animal, cell | KD, OE, AAV-shRNA | None | None |
| **Myocardial fibrosis** | Li et al., 2024 | *BCL-XL* mRNA (orthogonally verified site) | mRNA stability↑ → BCL-XL↑ →Caspase3↓ → cardiac fibroblasts apoptosis↓ → fibrosis | acRIP-qPCR, dual luciferase, actinomycin D, Remodelin (pharmacological only) | Dual luciferase | Animal, cell | None | Remodelin | None |
| **Periodontitis** | Liao et al., 2024 | *KLF6* mRNA (orthogonally verified site) | mRNA stability↑ →  pro-inflammatory | acRIP-qPCR, KD, OE, ropinirole, dual luciferase, molecular docking, KLF6 rescue | Dual luciferase | Cell | KD, OE | Ropinirole | None |
| **Periodontitis** | Zhang et al., 2023 | *NOX2* mRNA (candidate site) | NOX2↑→ROS↑→NF-κB↑ → pro-inflammatory | RNA-seq, KD, OE, Remodelin | None | Animal, cell | KD, OE | Remodelin | RNA-seq |
| **RA** | Liu et al., 2024 | *PTX3* mRNA (orthogonally verified site) | mRNA stability↑ → FLS migration/invasion↑ + immune infiltration (Dendritic cells, B cells, T cells)↑→pro-inflammatory | ac4C-RIP-seq, RNA-seq, acRIP-qPCR, dual luciferase, in vitro KD, in vivo haploinsufficiency, Adv-NAT10 OE | Dual luciferase | Human, animal, cell | In vitro KD, in vivo  Haploinsufficiency, Adv-NAT10 OE | Remodelin | ac4C-RIP-seq, RNA-seq |
| **IBD** | Li et al., 2025 | *Bag3* mRNA (candidate site) | mRNA stability↑ → T cell apoptosis↓ → CD4+ T cell pathogenicity maintenance ↑ → IBD | scRNA-seq, NAT10 RIP-qPCR, actinomycin D, RNA-seq, ATAC-seq | None | Human, animal, cell | cKO | None | scRNA-seq, RNA-seq, |
| **SLE** | Guo et al., 2020 | *Usp18/Gpx1/ Rgl1* mRNA (candidate sites) | May be involved in SLE pathogenesis (based on indirect evidence) | ac4C-RIP-seq, RNA-seq, LC-MS/MS (global ac4C quantification), acRIP-qPCR | None | Human | None | None | ac4C-RIP-seq, RNA-seq |
| **AD** | Ji et al., 2025 | *GRIN1/MAP2/ DNAJC6* mRNA (candidate sites) | ac4C↓ → translation efficiency↓ → synaptic protein insufficiency → early AD pathology | acRIP-qPCR, acRIP-seq, RNA-seq, Proteomics | None | Animal | None | None | acRIP-seq, RNA-seq |
| **AD** | Ma et al., 2023 | 37 mRNAs (candidate sites) | Differential mRNA ac4C modifications ↓ →ac4C enriched in GABAergic synapse and PI3K/AKT pathways → AD | ac4C-RIP-seq, RNA-seq, bioinformatic prediction | None | Animal | None | None | ac4C-RIP-seq, RNA-seq |
| **AD** | Ma et al., 2022 | lncRNAs (Gm26508, A430046D13Rik/9530059O14Rik) (candidate sites) | Differential lncRNA ac4C modifications → AD | ac4C-RIP-seq, RNA-seq, bioinformatic prediction | None | Animal | None | None | ac4C-RIP-seq, RNA-seq |
| **PWMI** | Li et al., 2025 | *Lipin1* mRNA (orthogonally verified site) | Lipin1 mRNA↓ → Lipin1 protein↓ → aberrant myelin development +impaired oligodendrocyte precursor cell differentiation | Point mutation, RNA pull-down, actinomycin D, KD, OE, Lipin1 rescue | Point mutation | Animal, cell | In vivo KD, OE, in vitro siRNA KD | None | None |
| **GBM** | Li et al., 2026 | *BOC* mRNA (orthogonally verified site) | HIF1α→NAT10 (transcriptional activation) → mRNA stability↑ +translation efficiency↑ →GBM proliferation/ migration | RNA-seq, acRIP-seq, acRIP-qPCR, NAT10 RIP-qPCR, RNA pull-down, actinomycin D, polysome profiling, point mutation, EIF3A RIP, KD, OE, Remodelin, HIF1α upstream validation | Point mutation | Human, animal, cell | KD, OE | Remodelin | RNA-seq, acRIP-seq |
| **GBM** | Wu et al., 2026 | R-loop  (Non-mRNA) | ac4C deposition→ stabilizes promoter R-loops→ maintains open chromatin→ *EGR1* transcriptional activation → GSC self-renewal →GBM proliferation / migration | R-loop IP-MS, RR-ChIP-seq, ac4C-RIP-seq, spatial transcriptomic, single-cell transcriptomic, KD, point mutation (WT/G614E rescue), EGR1 rescue, RNA stability, Remodelin | Point mutation | Animal, cell | KD | Remodelin | RR-ChIP-seq, ac4C-RIP-seq, RNA-seq, spatial transcriptomics, scRNA-seq |
| **RB** | Xu et al., 2025 | *HK1* mRNA (orthogonally verified site) | mRNA stability↑→ glycolysis↑ | ac4C RIP-qPCR, actinomycin D, dual luciferase, HK1 rescue, KD | Dual luciferase | Human, animal, cell | KD, | None | None |
| **RB** | Li et al., 2026 | *PFKFB3* mRNA (orthogonally verified site) | mRNA stability↑ → glycolytic flux↑ | ac4C RIP-qPCR, actinomycin D, dual luciferase, PFKFB3 rescue, KD, OE, 2-DG | Dual luciferase | Human (database), animal, cell | KD, OE | None | None |
| **GC** | Qian et al., 2026 | *DUSP1/FOSB* mRNA (orthogonally verified sites) | ①mRNA stability↑ → DUSP1↑→JNK/ERK apoptosis inhibition→ apoptosis evasion; ②FOSB↑→PD-L1↑ → immune escape↑ | RNA-seq, acRIP-qPCR, RNA pull-down, actinomycin D, dual luciferase, DUSP1 rescue, KD, OE, Remodelin, anti-PD-1 | Dual luciferase | Human, animal, cell | KD, OE | Remodelin, anti-PD-1 | RNA-seq |
| **GC** | Wang et al., 2026 | *HK2* mRNA (candidate site) | ac4C↓ → *HK2* mRNA stability / translation↓ → Glycolysis ↓ →inhibit GC | RNA-seq, acRIP-seq, acRIP-qPCR, Actinomycin D, OE, KD, HK2 rescue, 2-DG, Remodelin, GNE-493 | None | Animal, cell | OE, KD | Remodelin, GNE-493 | RNA-seq, acRIP-seq |
| **ICC** | Lu et al., 2026 | *CHAF1A* mRNA (orthogonally verified site) | mRNA stability↑ → CHAF1A↑→ silencing of HERV9NC →dsRNA↓ →innate immunity↓ → CD8+ T cell infiltration↓ →immune evasion | ac4C RIP-seq, RNA-seq, ATAC-seq, proteomics, UPLC-MS/MS, KD, OE, CHAF1A rescue, Remodelin, Panobinostat, scRNA-seq | UPLC-MS/MS | Human (tissue microarray), animal, cell | KD, OE | Remodelin, Panobinostat | ac4C RIP-seq, RNA-seq, ATAC-seq, scRNA-seq |
| **NSCLC** | Wang et al., 2024 | GAS5 lncRNA (candidate site) | mRNA stability↑ →MYBBP1A-p53-IRF1 →type I IFN → CXCL10 /CCL5→macrophage/T cell infiltration↑ →anti-tumor immunity↑ | RNA-seq, RNA pull-down+MS, ac4C RIP-qPCR, actinomycin D, GAS5 KD/OE, IRF1 KD/OE, NAT10 KD, IRF1 promoter dual luciferase, neutralizing antibodies, neoadjuvant immunotherapy cohort (pCR/DCB correlation analysis) | None | Human, animal, cell | GAS5 KD/OE, IRF1 KD/OE, NAT10 KD, neutralizing antibodies | Clinical correlation (not drug intervention) | RNA-seq |

**Table note:** Only core validation methods are listed; routine experiments (e.g., RT-qPCR, Western blot) are omitted. Unless otherwise specified, OE (overexpression), KD (knockdown), KO (knockout), and cKO (conditional knockout) all refer to manipulation of the NAT10 gene. “Perturbation experiments” include KD/KO/OE; “Treatments” refer to NAT10-targeting agents (small-molecule inhibitors or PROTAC degraders); “Tissue models” include human (clinical specimens/tissue microarrays/databases), animal (mice/rats), and cell-based models. Target gene-specific rescue experiments are individually noted in the “Validation methods” column.
