# Supplementary material for: The NAT10/ac4C axis: bridging RNA epigenetics to human diseases
Source: Front Cell Dev Biol. 2026 Oct 28;14:1905147. doi: 10.3389/fcell.2026.1905147 (PMC13752856; doi:10.3389/fcell.2026.1905147)
Supplement: Supplementary file 3 [file Table3.docx]

**Supplementary Table S3: Summary of NAT10 inhibitors**

| **Agent** | **Type** | **Mechanism of Action** | **Characteristics** | **Disease Models** |
| --- | --- | --- | --- | --- |
| **Remodelin** | Small-molecule inhibitor | Initially reported to bind NAT10 catalytic pocket and inhibit acetyltransferase activity | Subsequent studies have characterized it as a cryptic assay interference chemotype: no direct evidence of binding to NAT10 active sites; does not affect intracellular ac4C levels; should not be considered a specific NAT10 inhibitor | Cancer; tauopathy |
| **Fludarabine** | Small-molecule inhibitor: FDA-approved drug repurposing | Binds NAT10 Ac-CoA binding pocket and inhibits acetyltransferase activity | Shows comparable or stronger inhibitory effects on NAT10 than Remodelin; NAT10 selectivity remains to be clarified | Acute myeloid leukemia |
| **NP1192** | Selective PROTAC degrader | Simultaneously binds NAT10 and an E3 ubiquitin ligase, inducing NAT10 degradation via the ubiquitin-proteasome system | ~70% NAT10 degradation in cervical cancer cells; superior to Remodelin (IC₅₀ 26.8% lower); very low toxicity to non-malignant tissues; currently administered via i.p. injection; further optimization of solubility and oral bioavailability is required | Cervical cancer, ovarian cancer, glioblastoma |
| **Quetiapine** | Small-molecule inhibitor: FDA-approved drug repurposing | Directly binds NAT10; suppresses ac4C modification; inhibits OXPHOS pathway | FDA-approved antipsychotic; specific binding sites on NAT10 have not been identified; given the CNS effects associated with its psychiatric use, the benefit-risk ratio of quetiapine in cancers requires careful evaluation | Head and neck squamous cell carcinoma |
| **GNE-493** | Small-molecule inhibitor | PI3K/mTOR dual inhibitor; directly binds NAT10; reduces ac4C modification on HK2 mRNA | Dual targeting (conventional PI3K/mTOR inhibitor + novel NAT10 inhibitor); off-target toxicity associated with PI3K/mTOR inhibition remains a major concern | Gastric cancer (cisplatin-resistant) |
| **G-749** | Small-molecule inhibitor | Does not directly inhibit enzymatic activity; disrupts NAT10-USP39 interaction, promoting NAT10 degradation via the ubiquitin-proteasome system | Ser-536 and His-537 are critical for NAT10 targeting; acts as a PPI inhibitor; ATP6V0E1 is a downstream target, but whether other NAT10 substrates are also affected requires further investigation | Esophageal squamous cell carcinoma |
| **Paliperidone /AG-401** | Small-molecule inhibitor: FDA-approved drug repurposing (paliperidone) + small molecule from Specs library (AG-401) | Direct NAT10 binding confirmed by ITC (isothermal titration calorimetry) | Osteosarcoma cells are more sensitive to paliperidone and AG-401 than to Remodelin; the binding sites and inhibitory mechanisms of the two inhibitors remain to be elucidated; the clinical efficacy and safety should be evaluated in further research and clinical trials | Osteosarcoma |
